# Posterior decompression and internal fixation in treatment of hypertrophy of posterior longitudinal ligament at C1-2 level accompanied with lower cervical spinal stenosis: A case report

**DOI:** 10.1097/MD.0000000000005600

**Published:** 2016-12-16

**Authors:** Huan Liu, Tao Wang, Hui Wang, Wen-Yuan Ding

**Affiliations:** a Department of Spine Surgery, The Third Hospital of Hebei Medical University; b Hebei Provincial Key Laboratory of Orthopedic Biomechanics, Shijiazhuang, China.

**Keywords:** HPLL, internal fixation, lower cervical spinal stenosis, posterior decompression

## Abstract

**Rationale:**

Hypertrophy of posterior longitudinal ligament (HPLL) at C1-2 level accompanied with lower cervical spinal stenosis is rare in clinic. No reports have described HPLL at C1-2 level accompanied with lower cervical spinal stenosis treated by posterior decompression, combined with internal fixation in 1 stage.

**Patient concerns:**

A 70-year-old Chinese female complained of numbness and paralysis in both her hands and right leg for 1.5 years; Cervical vertebra x-rays and magnetic resonance imaging revealed a HPLL at C1-2 and cervical spinal stenosis at C3-6.

**Diagnoses:**

She was diagnosed with cervical spondylotic myelopathy (CSM).

**Interventions:**

The patient underwent posterior decompression from C1 to C5 level, and fixed with C1-2 vertebral pedicle and C3-5 lateral mass of screw.

**Outcomes:**

One week after operation, the patient showed significant improvement in the numbness of her hands. A follow-up cervical vertebra computed tomography showed good location of internal fixation device and correction of cervical spinal stenosis. Twelve months after surgery, the patient showed improvement in preoperative clumsiness and gait disturbance, and no recurrence of the clinical symptoms occurred.

**Lessons:**

HPLL at C1-2 level accompanied with lower cervical spinal stenosis caused myelopathy is rare. Cervical posterior decompression and internal fixation is an effective treatment. The surgical outcome is satisfactory.

## 1 Introduction

Hypertrophy of posterior longitudinal ligament is a common disease which could cause the compression of the cervical spinal cord and lead to pain, postural difficulties, or other neurological symptoms.^[1]^ Cervical spinal stenosis is mostly due to degenerative change or trauma. It usually occurs below C3, and rarely occurs in upper cervical spinal.^[2]^ However, no reports have described HPLL at C1-2 level accompanied with lower cervical spinal stenosis, both of which require surgical treatment. Here, we present a unique instance of a female who suffered from HPLL at the C1-2 level accompanied with stenosis at C3-6, and underwent posterior decompression combined with C1-2 vertebral pedicle and C3-5 lateral mass of screw fixation.

### 1.1 Ethics statement

The current study was approved by Ethics Committee of the Third Hospital of Hebei Medical University, also known as Ethics Committee of Hebei Provincial Orthopedic Hospital. There is no need to obtain informed consent from the patient since all the data were collected and analyzed anonymously. Also, it was approved by Ethics Committee of the Third Hospital of Hebei Medical University, also known as Ethics Committee of Hebei Provincial Orthopedic Hospital.

## 2 Case report

**Figure 1:**
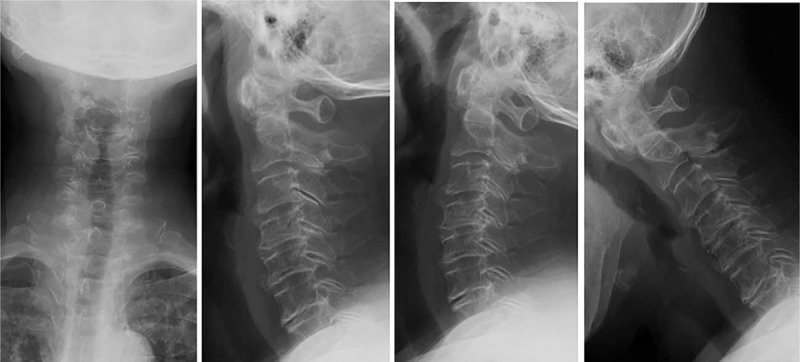
Plain cervical x-ray showed mild degenerative change without significant atlantoaxial instability.

**Figure 2:**
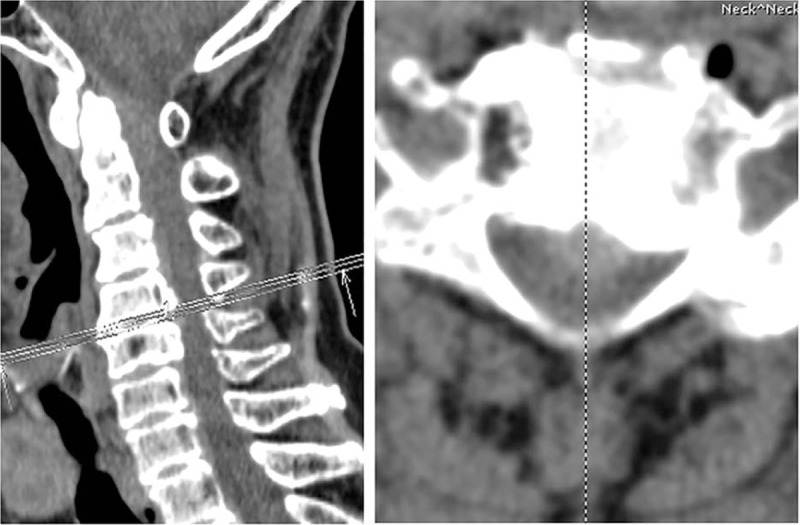
Cervical spinal computed tomography revealed stenosis of spinal canal at the lower cervical.

**Figure 3:**
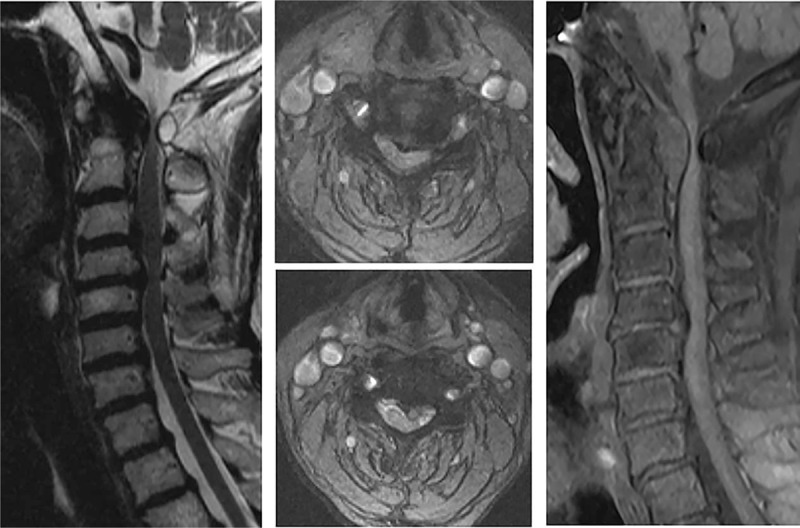
Magnetic resonance imaging and enhanced magnetic resonance imaging revealed hypertrophy of posterior longitudinal ligament at C1-2 and consecutive spinal canal stenosis from C3 to C6 levels. Spinal cord was compressed at both upper and lower level.

The patient was a 70-year-old Chinese female who complained of numbness and paralysis of her both hands and right leg for 1.5 years. She had type 2 diabetes for at least 5 years, no other major medical history was noted, and physical therapy and acupuncture therapy did not work. In physical examination, there were no motor or sensory deficits, except for the sensation of numbness in her both hands and right leg, and the muscle power of the both hands was 4/5. The Japanese Orthopaedic Association (JOA) score was 7. The bilateral Hoffmann sign was positive. The bilateral brachial II triceps, knee, ankle reflex active, and spastic gait was noted. Plain cervical x-ray and computed tomography (CT) showed mild degenerative change without significant atlantoaxial instability (Figs. 1 and 2). Magnetic resonance imaging (MRI) and enhanced MRI revealed HPLL at C1-2 and consecutive spinal canal stenosis from C3 to C6 levels (Fig. 3).

**Figure 4:**
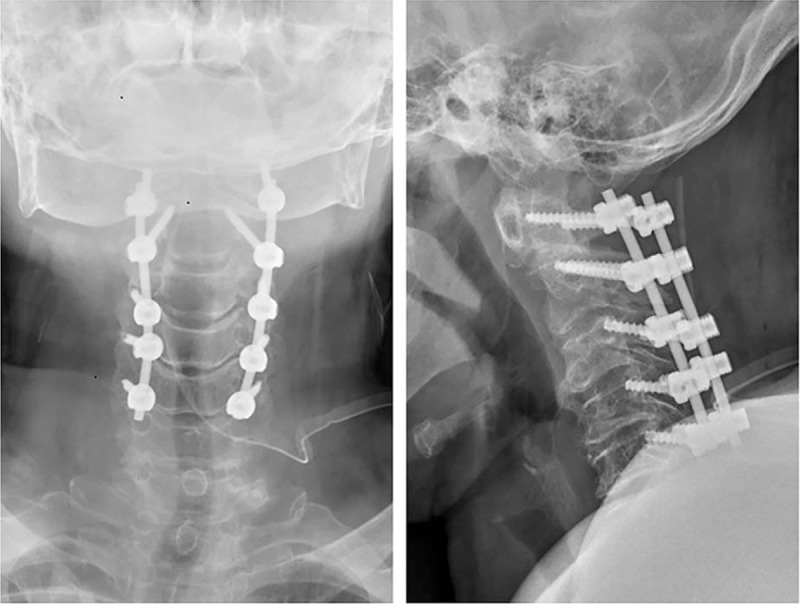
The postoperative x-ray showed good location of internal fixation device.

**Figure 5:**
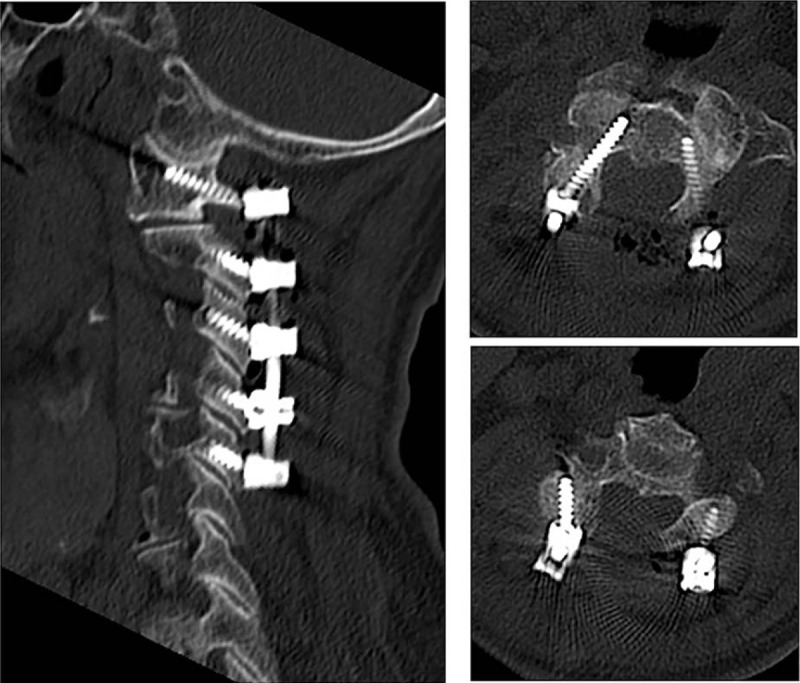
The postoperative computed tomography showed C1-2 vertebral pedicle and C3-5 lateral mass were fixated by posterior screw-rod system. The narrow canal had been corrected.

In July 2015, she underwent a posterior decompression combined with C1-2 vertebral pedicle and C3-5 lateral mass of screw fixation. Initially, removal of the posterior arch of the atlas and laminectomy from C2 to C5 was performed. Then, C1-2 vertebral pedicle and C3-5 lateral mass were fixated by posterior screw-rod system assisted by CT-based navigation system. X-ray and CT scan were performed at operated levels postoperatively to evaluate the accuracy of the screw insertion (Figs. 4 and 5). One week after operation, the patient showed significant improvement in the numbness of her hands, the spastic gait was improved 50%, the JOA score was 10, and the improvement rate was 30%, which suggested that the surgery is efficient. Twelve months after surgery, the patient showed improvement in preoperative clumsiness and gait disturbance, the JOA score was 13, the improvement rate was 60%, and no recurrence of the clinical symptoms occurred.

## 3 Discussion

Hypertrophy of posterior longitudinal ligament, which could transform into ossification of posterior longitudinal ligament (OPLL), is a major cause of spinal cord compression.^[3,4]^ The incidence of cervical spondylotic myelopathy (CSM) combined with degeneration of the posterior longitudinal ligament is 52%.^[5]^ Although cervical spinal stenosis is well known, cervical spinal stenosis at C1-2 accompanied with lower cervical spinal stenosis is extremely rare.^[6,7]^ More than a dozen patients with symptomatic isolated upper cervical spinal stenosis had been reported, but all of them were without lower cervical spinal stenosis. The author analyzed a patient who suffered from HPLL at upper cervical spinal accompanied with stenosis at lower level.

There are many possible causes involved in pathogenesis of HPLL. Firstly, genetic factor may play an important role. The incidence of HPLL in immediate relatives is far higher than the general population. Secondly, mechanical stress and cytokines may be associated with HPLL, and the proteins of fibroblasts in posterior longitudinal ligament may be expressed abnormally under mechanical stress. Additionally, some collagen diseases like rheumatoid arthritis (RA) may be associated with this kind of HPLL at C1-2. The pathological mechanism of RA patient's ligament and joint capsule tissue is inflammatory cell infiltration after the vasodilatation process, which is a process of chronic nonspecific inflammatory infiltration. The ligament tissue of RA patients is hypertrophic. Finally, the degeneration of posterior longitudinal ligament may be the pathogenetic basis of HPLL. The range of motion of atlantoaxial joint is greater than lower cervical spine. In this case, atlantoaxial overactivity may contribute to the compression of spinal cord and the development of symptoms.^[8–10]^

Adequate radiological examination is fundamental in diagnosis of the hypertrophy of posterior longitudinal ligament. Plain cervical x-ray revealed mild degenerative change without significant atlantoaxial instability. A cervical intervertebral disc CT scan showed stenosis of spinal canal and compression of spinal cord at C1-6. MRI showed abnormal signals behind odontoid and lower cervical spinal stenosis. Enhanced MRI is required to confirm the HPLL at C1-2 level.

Treatment is surgical decompression and fixation. In view of the multisegment compression of spinal cord, removal of the posterior arch of atlas and laminectomy from C2 to C5 were performed. The main mechanism of posterior decompression was to directly decompress the pressure and indirect decompression by postoperative spinal cord drift. In patients with cervical stenosis with myelopathy who underwent cervical laminectomy, posterior instrumentation has been demonstrated to reduce the incidence of postoperative instability and kyphosis.^[11]^ Therefore, C1-2 vertebral pedicle and C3-5 lateral mass were fixated by posterior screw-rod system assisted by CT-based navigation system. The case we have presented here who received surgical treatment demonstrated significant improvement after operation.

In conclusion, HPLL at C1-2 level accompanied with lower cervical spinal stenosis caused myelopathy is rare. Cervical posterior decompression and internal fixation is an effective treatment. The surgical outcome is satisfactory.
